# Impact of ultrasound assisted pretreatment and drying methods on quality characteristics of underutilized vegetable purslane

**DOI:** 10.1016/j.ultsonch.2024.107194

**Published:** 2024-12-09

**Authors:** Tajali Assad, Zahida Naseem, Sajad Mohd Wani, Aisha Sultana, Iqra Bashir, Tawheed Amin, Fauzia Shafi, B.S. Dhekale, Imtiyaz Tahir Nazki, Imtiyaz Zargar, A Raouf Malik, Tawfiq Alsulami, Robert Mugabi, Gulzar Ahmad Nayik

**Affiliations:** a Division of Food Science and Technology, Sher-e-Kashmir University of Agricultural Sciences and Technology of Kashmir, Shalimar, Srinagar, J&K 190025, India; b Division of Basic Science and Humanities, Sher-e-Kashmir University of Agricultural Sciences and Technology of Kashmir, Shalimar, Srinagar, J&K 190025, India; c Division of Agricultural Statistics, Sher-e-Kashmir University of Agricultural Sciences and Technology of Kashmir, Shalimar, Srinagar, J&K 190025, India; d Division of Floriculture and Landscape Architecture, Sher-e-Kashmir University of Agricultural Sciences and Technology of Kashmir, Shalimar, Srinagar, J&K 190025, India; e Division of Fruit Science, Sher-e-Kashmir University of Agricultural Sciences and Technology of Kashmir, Shalimar, Srinagar, J&K 190025, India; f Department of Food Science & Nutrition, College of Food and Agricultural Sciences, King Saud University, Riyadh 11451, Saudi Arabia; g Department of Food Technology and Nutrition, Makerere University, Kampala, Uganda; h Marwadi University Research Centre, Department of Microbiology, Marwadi University, Rajkot, Gujarat 360003, India

**Keywords:** Ultrasound, Drying methods, Purslane, Rehydration ratio, Antioxidant activity

## Abstract

The present study was aimed to determine the effect of ultrasound pretreatment and different drying methods viz sun drying, solar drying, cabinet drying, vacuum drying, microwave assisted drying and freeze drying on physicochemical, phytochemical activity, rehydration ratio and drying time of the purslane. The purslane was ultrasonicated for 15, 30, 45 and 60 min following by drying. The ultrasound pretreatment (60 min) combined with freeze drying retained the highest antioxidants (95.59 %), phenolic content (7.85 mgGAE/100 g), total carotenoid content (99.74 mg/100 g), ascorbic acid (399.94 mg/100 g) and rehydration ratio (6.80). Moreover, the same combination revealed higher *L* and a* values when compared with other drying methods. However, the purslane pretreated with ultrasonication for 60 min and then dried via microwave took less time for drying. This study suggests that Ultrasound pretreatment (60 min) followed by freeze drying is recommended for preserving the nutritional and functional properties of purslane. It could be scaled up for commercial applications in the functional food and nutraceutical industries, where high-quality preservation is crucial.

## 1 Introduction

Purslane (*Portulaca oleracea),* a herb which is more common in the Mediterranean region, particularly in the semi-arid and dry countries of Southern Europe and Northern Africa [1]. In India, it grows as a weed and reaches as high as 1500 m in the Himalayas. Additionally, it is grown as a vegetable. Purslane is a climate-resilient and underutilized crop, rarely grown on a commercial scale or widely traded [2]. Purslane is a potential crop for dietary diversification since cereal crops, which provide the majority of the world's food, are highly sensitive to climatic variations.

The nutritional, phytoremediation, therapeutic and pharmacological qualities of purslane are remarkable. It is very nutrient-dense and contains carbohydrates, protein, minerals (potassium, calcium, magnesium, phosphorus, iron) [3]. It also contains vitamin A, E, B complex (such as niacin, riboflavin, pyridoxine) and vitamin C. Purslane has four distinct kinds of omega-3 fatty acids. This is necessary for maintaining a healthy immune system, improving and preventing cardiovascular diseases, and overall welfare [4].

In addition to its culinary uses, purslane has been utilized as herbal remedy traditionally. It has been proven to have anti-inflammatory and analgesic qualities, as well as anti-cancer activity and antioxidant activity [5]. This herb can be applied externally to ease bug bites and treat a variety of skin conditions, including acne, boils, eczema and can also be used to treat fever, bronchitis and coughs [6].

Due to metabolic reactions, purslane is extremely perishable, can lose quality, weight, economic value and food value. Preventing these losses is very important, particularly when there is an imbalance in supply and demand during the off-season. It is necessary to protect nature's reservoir of nutrients by using different preservation methods. Purslane has been preserved using a variety of processing methods, including dehydration, canning, freezing, pickling, vacuum-packing and irradiation. Among the mentioned techniques, dehydration looks to be the sophisticated technology for protective such green leafy vegetables. Nonetheless, a number of drawbacks to dehydration techniques have been noted, including the comparatively high energy usage and product quality degradation associated with hot drying, uneven drying in microwave, high cost associated with freeze and hybrid drying, etc. Pretreatment is therefore, frequently used prior to drying.

Pre-treatment before drying is a thoroughly researched topic in which numerous techniques have been established. To date, various chemical and physical pretreatment techniques have been used for fruits and vegetables [7]. The chemical pretreatments can expedite the drying process, but they also deplete nutrients that are soluble and result in toxic residues that compromise food safety. Microorganisms can be eliminated by thermal pre-treatment, which also softens the texture and speeds up drying. Nevertheless, it results in unfavorable quality.

However, non-thermal technologies (such as pulsed electrical fields and ultrasound) may be a better option to get over these problems. In recent years, there has been a surge in the use of ultrasound pre-treatment for food products, which has the potential to significantly reduce drying period. The application of ultrasonic (US) technology not only decreased the overall drying duration and energy usage, but also improved the attribute of the final product. US aided drying is a cost-efficient and ecofriendly technique in comparison to other conventional methods now in use for food processing and preservation. The travel of ultrasonic waves through a medium lead to series of compression and rarefaction waves that is similar to sponge effect. The sponge effect occurs when ultrasonic waves travel through the fruit or vegetable, causing a rapid alternating compression and expansion of the fruit or vegetable tissue. This phenomenon was called the “sponge effect” because it can be compared to what happens when a sponge is squeezed and released multiple times [8]. It generates microscopic channels which facilitates removal of moisture. These microscopic channels are used by water molecules to move towards the surface of fruits and vegetables [9]. While prior studies have examined the impact of ultrasound-assisted pretreatment or individual drying methods on various food matrices, the combined effect of US-assisted pretreatment with multiple drying techniques remains underexplored.

Thus, the purpose of the current study was to ascertain how ultrasound pretreatment and various drying techniques, such as sun, solar, cabinet, microwave assisted, vacuum and freeze drying, affected the physicochemical properties, phytochemical activity, rehydration ratio, chlorophyll content, drying time, and color of purslane. Furthermore, the study will provide a cost effective and efficient method for drying of leafy vegetables.

## 2 Materials and methods

### 2.1 Preparation of sample

Fresh purslane (*Portulaca oleracea*) was harvested from the field of Sher-e-Kashmir University of Agricultural Science and Technology of Kashmir, Shalimar. The green leaves and soft stems were detached from plant and cleaned using water, spread on the tray which was covered with muslin cloth at room temperature in order to drain the water from it.

### 2.2 Chemicals and reagents

All chemicals and reagents like aluminum chloride, sodium hydroxide, Folin-Ciocalteu reagent, sodium nitrite, gallic acid, anhydrous sodium carbonate, DPPH used were from Hi Media (Mumbai). Methanol, hexane and acetone were of analytical grade.

### 2.3 Sonication

The purslane samples were immersed in ultrasonic equipment bath having capacity 3.2 L (Indosati, model INDO/AB/008) using distilled water as medium at a constant temperature of 20 °C and frequency of 40 kHz in dark to avoid any interference of light with samples and ultrasonic power of 120 W. To prevent contamination of the test samples, the ultrasonic tank was washed twice with sterile water and then with 70 % alcohol. Four different ultrasound treatments *viz* P1 (US treatment for 15 min), P2 (US treatment for 30 min), P3 (US treatment for 45 min), P4 (US treatment for 60 min), while as untreated sample acted as control (P0).

### 2.4 Drying process

After the ultrasound treatment, the purslane leaves were blotted with filter paper and subjected to drying. Drying of purslane was conducted using six different methods of drying *viz* sun drying (M1), solar tunnel drying (M2), cabinet drying (M3), vacuum drying (M4), freeze drying (M5) and microwave assisted drying (M6).

In sun drying, samples were spread in a single layer on trays and placed outdoors in direct sunlight. Drying was carried out till moisture content reached 5 ± 1 % (wb) and the temperature on average was about 25 ± 2 °C.

In solar drying, pretreated samples were spread in a single layer on trays and placed in solar drier (Rudra Solar Dryer), which was kept on ground at an angle of 30 degree. The size of solar dryer was 6ft × 3ft having tray size of 16˝×32˝ and voltage of solar panel was 30 W. Drying was carried at 50 °C till moisture content reached 5 ± 1 % (wb).

In cabinet drying, samples were dried in a hot air oven (Memmert, Model: UN55). Ultrasound pretreated samples were placed on trays in a thin layer and dried at 60 °C till moisture content reached 5 ± 1 % (wb).

Vacuum drying was performed in a Vacuum Oven (WiseVen, model WOV – 30, Japan) with internal dimensions of the oven cavity 300 × 330 × 300 mm. Samples were dried (at 60 °C) till moisture content reached 5 ± 1 % (wb) and during the drying process pressure was maintained at 200 mmhg.

Freeze drying was carried in a freeze-drier (Wiswo Cap. 5–6 Ltr. Sr. No: 548/508). Samples were kept in a deep freezer at –40 °C. Frozen samples were then transferred to petri dishes and kept in freeze dryer till moisture content reached 5 ± 1 % (wb).

Microwave assisted drying was carried in microwave assisted drier (Model no. TW/MWVAC/BATCH/012). The Purslane samples were dried at 60 °C till moisture content reached 5 ± 1 % (wb) and during the drying process pressure was maintained at 500 mmhg.

### 2.5 Characterization of the dried samples

The US pretreated samples dried by six different drying methods were evaluated for various parameters.

#### 2.5.1 Drying time

Total drying time for different pretreatments and drying methods was calculated by using the following equation

(1) $$t=\frac{\ln(Mo/M)}{0.396}$$

where, Mo is initial moisture content and M is final moisture content

#### 2.5.2 Moisture content

Moisture content of dried purslane was estimated according to [10]. The samples were heated in a convection oven at 105 °C till constant weight. Moisture content of samples was determined as per the Eq. (1).

(2) $$\mathrm{moisture}\;content\;\left(\%\right)=\frac{\mathrm{weight}\;loss\;(g)}{\mathrm{weight}\;of\;sample\;}\times 100$$

#### 2.5.3 Water activity

Water activity was measured by a water activity meter (Aqualab) at 25 °C ± 0.5.

#### 2.5.4 Rehydration ratio

Rehydration ratio was estimated by the method of [11]. The dried sample was soaked in distilled water for 9 h at 50 °C. Samples were taken out at intervals of 1 h and weighed after being gently drying the sample surface using filter paper. RR was calculated from the following equation

(3) $$\mathrm{Rehydrationratio}\left(RR\right)=\frac{Mr\;}{\mathrm{Md}}$$

where M_r_ is the weight of wet samples and M_d_ is the initial weight of the dry sample.

#### 2.5.5 Crude fat content

The crude fat of the dried purslane was estimated by the method approved by [12].

#### 2.5.6 Chlorophyll content

Chlorophyll content was estimated by the method outlined by [13]. The procedure involved mixing 99 % acetone and ethanol in a 2:1 vol ratio (25 ml total) using a volumetric flask. Subsequently, dried samples were immersed in the solution for 24 h and the volumetric flask was shaken every 4 h during this period. Afterward, absorbance was taken at wavelengths of 645 and 663 nm and the chlorophyll content was estimated by the following equation

(4) ca=12.72A663-2.59A645

cb=22.88A645-4.67A663

ctotal=ca+cb

where ca is chlorophyll *a*, cb is chlorophyll *b*, ctotal is the total chlorophyll content, A663 and A645 are the absorptions of the extract at 663 and 645 nm

#### 2.5.7 Carotenoid content

Total carotenoid was estimated by the method of [14] with little modifications. 5 g of dry samples were mixed in cold acetone, vortexed and left for 2 h in refrigeration till the residue was colorless. The extract was filtered. Acetone extract (25 mL) was transfered into a separating flask and petroleum ether (20 mL) was added and the contents were kept as for 15 min. Then 5 % NaCl solution (100 mL) was added and the content was blended properly and allowed to separate into two phases and the organic phase was collected. The organic phase was cleaned many times with deionized water to get rid of residual acetone. Remaining water was eliminated from the petroleum ether phase by running it via funnel filled with sodium sulphate (anhydrous) before collecting it in a volumetric flask. After adding petroleum ether to the contents to make a 100 mL volume, the absorbance was taken at 450 nm. The following formula was used to determine the total carotenoids as mg β-carotene equivalents/100 g:

(5) Totalcarotenoid(mg/100g)=(ΔA/€L)×MW×D×(V/G)

where ΔA is absorbance, € molar extinction coefficient of beta-carotene (2590), L length of cell path (1 cm), MW the molecular weight of beta-carotene (536.8), D a dilution factor, V the final volume (mL) and G weight of sample (g).

#### 2.5.8 Ascorbic acid content

Ascorbic acid was calculated following the method of [15]. The dried sample was mixed in 3 % metaphosphoric acid solution and titrated against 2,6-dichlorophenol indophenol dye till light pink color appears.

#### 2.5.9 Ash and mineral content

The ash content was determined by ashing the dried sample (5 g) in a muffle furnace (Toshiba). The mineral content was estimated by dissolving the resulting ash in 5 ml of HCl and analyzed using the atomic absorption spectrophotometer (AAS model, SP9).

#### 2.5.10 Antioxidant activity

The method outlined by [16] was used to evaluate radical scavenging activity, which uses the free radical 2,2-diphenyl-l-picrylhydrazyl (DPPH). Methanolic extract 0.1 ml was added to 3.9 ml of a 0.01 % methanolic DPPH solution. The UV–Vis spectrophotometer (IG-28DS) was used to measure the absorbance at 517 nm after 30 min. The Eq. 6 was used to compute the results as the percent inhibition

(6) $$\%inhibition=\frac{\mathrm{Absorbance}\;of\;control\left(517\right)-Absorbance\;of\;sample\left(517\right)}{\mathrm{Absorbance}\;of\;control(517)}\times 100$$

#### 2.5.11 Color (L*, a* and b*)

Color (L*, a* and b* value) of dried purslane was determined by HunterLab Colorimetri (ColorFlex EZ). The device was calibrated using the calibration scale before color measurement [17].

#### 2.5.12 Total phenolic content(mgGAE/g)

The total phenolic content was calculated using the method described by [18]. An eppendorf tube was filled with distilled water (7.9 ml) methaolic extract (0.1 ml) and Folin-Ciocalteu reagent (0.5 ml) (1:1 with water), then1.5 ml of sodium carbonate (20 %) was added after one minute, and the mixture was thoroughly shaken and was kept in the dark for two hours, then the absorbance at 765 nm was measured. Gallic acid was used as the reference in the calculation of the total phenolic concentration, and the outcome was reported as milligrammes of gallic acid equivalents/100 grammes (mgGAE/100 g).

#### 2.5.13 Total flavonoid content (mgQE/g)

Total Flavonoid (mgQE/ g) was estimated according to [19]. The purslane extract (100 μl) and sodium nitrate (5 % w/v) (100 μl) were mixed, incubated for 6 min. Then aluminum chloride (10 % w/v) (150 μl) was added and rested for 5 min, then NaOH (1 M) (200 μl) was added sequentially. The absorbance at λ 510 nm on UV–Vis spectrophotometer (IG-28DS) was measured. A standard quercetin curve was used as the comparison.

#### 2.5.14 Statistical analysis

The obtained data was subjected to statistical analysis using the SPSS statistics software (v.250 16, Inc., Chicago, IL) through one-way analysis of variance (ANOVA) at 5 % level of significance. To determine significant differences between the means, the Duncan's multiple range test (DMRT) was employed.

## 3 Results and discussion

### 3.1 Drying time, moisture content (%) and water activity

The effect of ultrasound pre-treatments and different drying methods on the moisture content, water activity and drying time of purslane samples was studied and results are presented in Table 1. Moisture content is an essential parameter that influences the quality and shelf-life of product. Different pretreatments and dehydration processes caused no significant changes (*p* ≥ 0.05) in moisture content in all the purslane samples. The moisture content of untreated dried purslane samples was between 5.60–6.55 % and for ultrasound pretreated samples, it ranged from 4.59-6.30 %. P0M1 samples had highest moisture level and the P4M5 samples had the lowest moisture content. Shams *et al*., [20] also found similar results and concluded that compared to other drying methods, freeze drying resulted in a greater loss of water. Water activity (a_w_) determines the physical and chemical stability of product during storage. Lower water activity means long shelf life. Different pretreatments caused no significant (*p* ≥ 0.05) changes while different drying methods caused significant (*p* ≤ 0.05) changes in water activity in all the purslane samples which were give ultrasound pretreatment. The water activity of untreated samples was in range of 0.30–0.49 while it ranged from 0.22 to 0.49 in the samples which were given ultrasound pre-treatment. The water activity was low in the sample pretreated with ultrasound for 60 min followed by freeze drying while the a_w_ found in untreated sun-dried sample was high. The results of present findings are in line with [21], who observed decrease in water activity of barley grass pretreated with ultrasound. Shams *et al*., [20] also found that the moisture content and water activity in freeze drying were lower and attributed it to the high removal of moisture content as a result of sublimation. The drying time of untreated dried purslane samples were in range of 80–1620 min while it ranged from 48 to 1178 min in the samples pretreated with ultrasound following by drying. The maximum drying time (1620 min) of purslane sample was recorded in the untreated sample which was sun dried while the minimum drying time (48 min) was recorded in the sample which was given ultrasound pretreatment (60 min) combined with microwave assisted drying. The outcome of this study is in line with the results of [22] and [23], they reported that ultrasound pretreatment and microwave vacuum drying reduced the drying time by 27.3 % and 10 % respectively. The decrease in moisture content and water activity while decrease in drying time might be due to the microscopic channels and pores generated by the ultrasound pretreatment within the food structure, which facilitate faster water movement from the interior to the surface, thereby accelerating moisture diffusion. These microchannels increase the exposed surface area and reduce resistance to water transfer during drying. Additionally, ultrasound-induced vibrations and agitation break down the boundary layer of water vapor surrounding the food particles, enhancing mass transfer rates. This reduction in boundary layer thickness also improves convective heat and mass transfer rates, further shortening drying time.

**Table 1:** Effect of ultrasound (US) pretreatment and different methods on the drying time, Moisture content, water activity and rehydration ratio of Purslane.

		P0	P1	P2	P3	P4
Drying time (minutes)	M1	1620 ± 0.07^aA^	1178 ± 0.09^aB^	1053 ± 0.02^aC^	1020.6 ± 0.11^aD^	972 ± 0.10^aE^
	M2	0960 ± 0.08^cA^	662.4 ± 0.07^cB^	0624 ± 0.15^cC^	604.8 ± 0.09^cD^	576 ± 0.09^cE^
	M3	0240 ± 0.11^eA^	165.6 ± 0.04^dB^	0156 ± 0.08^dC^	151.2 ± 0.10^dD^	144 ± 0.03^eE^
	M4	1380 ± 0.06^bA^	952.2 ± 0.10^bB^	0897 ± 0.09^bC^	869.4 ± 0.19^bD^	828 ± 0.06^bE^
	M5	0940 ± 0.08^dA^	662.4 ± 0.11^cB^	0624 ± 0.12^cC^	604.8 ± 0.14^cD^	564 ± 0.05^dE^
	M6	0080 ± 0.09^fA^	055.2 ± 0.04^eB^	0052 ± 0.07^eC^	050.4 ± 0.13^eD^	48 ± 0.08^fE^
Moisture content (%)	M1	6.55 ± 0.26^aA^	6.30 ± 0.28^aA^	5.76 ± 0.54^aA^	5.68 ± 1.10^aA^	5.60 ± 0.41^aA^
	M2	6.27 ± 0.71^aA^	6.09 ± 1.04^aA^	5.88 ± 0.38^aA^	5.44 ± 0.74^aA^	5.32 ± 0.72^aA^
	M3	5.55 ± 0.75^aA^	5.42 ± 0.09^aA^	5.35 ± 1.19^aA^	5.27 ± 0.43^aA^	5.11 ± 0.73^aA^
	M4	5.69 ± 0.49^aA^	5.50 ± 0.91^aA^	5.36 ± 0.99^aA^	5.18 ± 0.70^aA^	5.09 ± 0.73^aA^
	M5	5.30 ± 0.19^aA^	5.18 ± 0.81^aA^	5.12 ± 1.41^aA^	5.02 ± 0.51^aA^	4.59 ± 1.67^aA^
	M6	5.42 ± 0.86^aA^	5.31 ± 0.20^aA^	5.22 ± 0.58^aA^	5.15 ± 0.78^aA^	5.01 ± 0.65^aA^
Water activity	M1	0.49 ± 0.13^aA^	0.49 ± 0.06^aA^	0.46 ± 0.09^aA^	0.42 ± 0.02^aA^	0.39 ± 0.05^aA^
	M2	0.48 ± 0.08^abA^	0.45 ± 0.13^abA^	0.42 ± 0.07^abA^	0.38 ± 0.13^abA^	0.35 ± 0.13^aA^
	M3	0.45 ± 0.09^abA^	0.41 ± 0.09^abcA^	0.35 ± 0.07^abA^	0.34 ± 0.07^abA^	0.32 ± 0.12aA
	M4	0.36 ± 0.03^abA^	0.32 ± 0.02bcA	0.31 ± 0.14^abA^	0.29 ± 0.05^abA^	0.26 ± 0.05^aA^
	M5	0.30 ± 0.12^bA^	0.29 ± 0.03^bA^	0.27 ± 0.08^cA^	0.23 ± 0.09^bA^	0.22 ± 0.10^aA^
	M6	0.33 ± 0.10^abA^	0.31 ± 0.08^bcA^	0.28 ± 0.10^abA^	0.27 ± 0.05^abA^	0.24 ± 0.07^aA^
Rehydration ratio	M1	3.47 ± 0.06^eC^	3.64 ± 0.17^eB^	3.67 ± 0.05^fB^	3.69 ± 0.10^fB^	4.38 ± 0.05^eA^
	M2	3.70 ± 0.04^dE^	3.98 ± 0.09^dD^	4.41 ± 0.06^eC^	4.86 ± 0.06^eB^	5.77 ± 0.08^dA^
	M3	4.20 ± 0.08^cD^	4.72 ± 0.05^bC^	5.30 ± 0.08^cB^	5.99 ± 0.09^cA^	6.10 ± 0.05^cA^
	M4	3.80 ± 0.03^dE^	4.40 ± 0.11^cD^	5.43 ± 0.11^bC^	6.30 ± 0.07^bB^	6.45 ± 0.07^bA^
	M5	5.12 ± 0.07^aD^	5.64 ± 0.08^aC^	5.91 ± 0.09^aB^	6.67 ± 0.11^aA^	6.80 ± 0.08^aA^
	M6	4.50 ± 0.07^bE^	4.77 ± 0.09^bD^	5.02 ± 0.08^dC^	5.60 ± 0.16^dB^	6.78 ± 0.09^aA^

Values are expressed as Mean ± SD (n = 3), a-f within a column and A-F within a row, with different letters are significantly different. M1 (sun drying), M2 (solar tunnel drying), M3 (cabinet drying), M4 (vacuum drying), M5 (freeze drying) and M6 (microwave assisted drying). P0 (control), P1 (US treatment for 15 min), P2 (US treatment for 30 min), P3 (US treatment for 45 min), P4 (US treatment for 60 min).

### 3.2 Rehydration ratio

Rehydration defines most important quality characteristics for drying foods and also highlights the physicochemical alterations brought by pre-treatments, sample composition and process conditions during drying [24]. The high rehydration ratio indicates the quality of the dried product since the pores permit water to re-enter the cells. The effect of ultrasound pre-treatments and different dehydration methods on the rehydration ratio (RR) of purslane samples is given in Table 1. Different pretreatments and dehydration processes caused significant (*p* ≤ 0.05) changes in rehydration ratio in all the purslane samples. From the Table 1, it is observed that the rehydration ratio of untreated dried purslane samples ranged between 3.47–5.12 while for treated and dried samples, it ranged from 3.64 to 6.80. The highest RR was observed in P4M5, while the lowest was in P0M1 samples. This higher RR could be due to porous structure caused by ultrasound. The higher porosity and micro-channel formation allowed the better entrance of water during rehydration. The present study is well supported by [25], who studied the mechanism involved in enhancement of rehydration process of carrot slices caused by the pretreatment using the ultrasound technology. They found that the rehydration of samples subjected to ultrasonic pre-treatment at both 30 and 60 min was greater compared to untreated samples, which can be attributed to the higher porosity. The outcomes are also in accordance with the studies of [26] and [27] who investigated the effect of different drying methods on rehydration ratio and concluded that freeze drying has better RR value.

### 3.3 Crude fat (%)

The impact of ultrasound pre-treatments and different drying methods on the crude fat of purslane samples was studied and the results are shown in Table 2. Different pretreatments and dehydration processes caused non-significant (*p* ≥ 0.05) changes in crude fat in all the purslane samples. The crude fat ranged from 3.30-3.35 % for untreated and for ultrasound pretreated samples, it ranged from 3.16-3.34 %. The crude fat in the P0M5 sample was higher than the P4M1.These phenomena can be correlated with the higher lipid oxidation and destruction or conversation of fat molecules in the other form due to ultrasound treatment and drying process. The reduction in fat content reduces the rancidity during storage; however, this could compromise the quality of the final product. The highest fat content in freeze drying could be owing to the absence of oxidizing agents. Lapena *et al*., [28] showed that there was no statistical effect on fat content with ultrasound pretreatment. Our results are in line with [29], [30] and [31].

**Table 2:** Effect of ultrasound (US) pretreatment and different methods on the Fat content, chlorophyll, Total carotenoid content and Ascorbic acid of Purslane.

		P0	P1	P2	P3	P4
Fat Content (%)	M1	3.20 ± 1.07^aA^	3.19 ± 0.21^aA^	3.18 ± 0.98^aA^	3.17 ± 0.91^aA^	3.16 ± 0.75^aA^
	M2	3.23 ± 0.73^aA^	3.22 ± 0.36^aA^	3.20 ± 1.02^aA^	3.19 ± 0.69^aA^	3.17 ± 1.61^aA^
	M3	3.26 ± 0.71^aA^	3.25 ± 0.86aA	3.24 ± 0.48^aA^	3.22 ± 0.95^aA^	3.20 ± 0.92^aA^
	M4	3.32 ± 0.63^aA^	3.31 ± 0.95^aA^	3.29 ± 0.71^aA^	3.28 ± 0.80^aA^	3.27 ± 0.90^aA^
	M5	3.35 ± 0.08^aA^	3.34 ± 0.72^aA^	3.32 ± 0.24^aA^	3.30 ± 0.45^aA^	3.28 ± 1.23^aA^
	M6	3.33 ± 1.37^aA^	3.32 ± 0.65^aA^	3.3 ± 0.70^aA^	3.29 ± 1.62^aA^	3.27 ± 0.60^aA^
Chlorophyll (mg/g)	M1	5.05 ± 0.05^dD^	5.44 ± 0.06^dC^	5.99 ± 0.06^eB^	6.24 ± 0.06^dA^	6.24 ± 0.06^eA^
	M2	5.33 ± 0.09^bE^	5.97 ± 0.11^cD^	6.30 ± 0.06^dC^	6.99 ± 0.02^cB^	7.15 ± 0.11^dA^
	M3	5.20 ± 0.07^cE^	5.98 ± 0.10^cD^	6.46 ± 0.07^cC^	7.00 ± 0.08^cB^	7.93 ± 0.05^cA^
	M4	4.78 ± 0.08^eD^	4.86 ± 0.09^eD^	5.01 ± 0.03^fC^	5.57 ± 0.05^eB^	5.78 ± 0.17^fA^
	M5	8.84 ± 0.06^aE^	8.99 ± 0.06^aD^	9.97 ± 0.04^aC^	10.24 ± 0.07^aB^	12.39 ± 0.08^aA^
	M6	5.32 ± 0.08^bE^	6.92 ± 0.11^bD^	7.51 ± 0.11^bC^	8.98 ± 0.06^bB^	9.47 ± 0.08^bA^
Total carotenoid content (mg/100 g)	M1	37.70 ± 0.14^fE^	44.56 ± 0.11^eD^	45.28 ± 0.08^eC^	46.01 ± 0.12^eB^	47.18 ± 0.05^fA^
	M2	42.86 ± 0.06^eE^	43.14 ± 0.10^fD^	43.98 ± 0.06^fC^	45.67 ± 0.04^fB^	49.78 ± 0.06^eA^
	M3	50.34 ± 0.08^dE^	53.27 ± 0.06^dD^	54.00 ± 0.060^dC^	55.45 ± 0.07^dB^	55.98 ± 0.05^dA^
	M4	53.88 ± 0.04^cE^	57.5 ± 0.05^bD^	58.61 ± 0.06^cC^	63.59 ± 0.11^cB^	66.9 ± 0.02^cA^
	M5	67.93 ± 0.03^aE^	79.26 ± 0.06^aD^	83.16 ± 0.08^aC^	94.05 ± 0.05^aB^	99.74 ± 0.06^aA^
	M6	55.81 ± 0.13^bE^	57.08 ± 0.06^cD^	62.71 ± 0.10^bC^	71.25 ± 0.09^bB^	79.48 ± 0.08^bA^
Ascorbic acid (mg/100 g)	M1	302.36 ± 0.10^fE^	302.58 ± 0.06^fD^	302.78 ± 0.08^fC^	302.95 ± 0.17^fB^	303.20 ± 0.09^fA^
	M2	320.12 ± 0.08^eE^	320.25 ± 0.04^eD^	320.56 ± 0.12^eC^	321.46 ± 0.08^eB^	321.98 ± 0.11^eA^
	M3	325.23 ± 0.05^dE^	325.48 ± 0.04^dD^	325.64 ± 0.14^dC^	326.48 ± 0.09^dB^	326.94 ± 0.07^dA^
	M4	336.20 ± 0.08^cD^	336.45 ± 0.06^cC^	336.49 ± 0.10^cC^	336.99 ± 0.08^cB^	337.56 ± 0.07^cA^
	M5	398.60 ± 0.04^aE^	398.92 ± 0.09^aD^	399.20 ± 0.07^aC^	399.49 ± 0.07^aB^	399.94 ± 0.08^aA^
	M6	340.41 ± 0.05^bD^	340.52 ± 0.05^bD^	340.98 ± 0.09^bC^	341.15 ± 0.04^bB^	341.96 ± 0.07^bA^

Values are expressed as Mean ± SD (n = 3), a-f within a column and A-F within a row, with different letters are significantly different. M1 (sun drying), M2 (solar tunnel drying), M3 (cabinet drying), M4 (vacuum drying), M5 (freeze drying) and M6 (microwave assisted drying). P0 (control), P1 (US treatment for 15 min), P2 (US treatment for 30 min), P3 (US treatment for 45 min), P4 (US treatment for 60 min).

### 3.4 Chlorophyll content

Green color of fruits and vegetables is due to the presence of chlorophyll, which is prone to thermal processing [32]. Pheophytin, a chlorophyll derivative serves as an indicator of chlorophyll degradation under specific conditions, such as heat or acid exposure. Thermally processed green vegetables undergo chlorophyll degradation and formation of a pheophytin or derivatives. Additionally, the amount of chlorophyll in leaves varies according to type, growing environment, and exposure to sunlight, which is an, essential component for the synthesis of chlorophyll. The effect of ultrasound pre-treatments and different drying methods on the chlorophyll content of purslane samples was evaluated. The chlorophyll content of untreated dried purslane samples was in range of 4.78–8.84 mg/g while for ultrasound pretreated samples dried with different methods, it varied from 4.86-12.39 mg/g (Table 2). The highest chlorophyll content was found in P4M5 samples. This may be attributed to reduced exposure to factors like heat, oxidation, and enzymatic activity that typically cause chlorophyll breakdown. By shortening drying time and enabling lower drying temperatures, thermal degradation is minimized, preserving the green color associated with chlorophyll. It also improves mass transfer, accelerating the drying process and reducing oxidation, which helps protect chlorophyll from oxidative damage. Furthermore, ultrasound can inactivate enzymes like chlorophyllase, which degrade chlorophyll, by disrupting cell membranes, thereby maintaining chlorophyll integrity in the leaves. Additionally, freeze drying immobilizes water, maintains enzyme inactivity thus retains more chlorophyll compared to other methods. Vargas *et al*., [33] reported that the temperatures exposure above 39.4 ◦C produced undesirable color changes in green peas due to decomposition of chlorophyll and other pigments, and also non-enzymatic browning. Our results are in line with the results of [34] and [35], who studied the impact of ultrasound and steam blanching pre-treatments on the drying kinetics, energy consumption and selected properties of parsley leaves and power ultrasound as a pretreatment to convective drying of mulberry (*Morus alba* L.) leaves: Impact on drying kinetics and selected quality properties respectively.

### 3.5 Total carotenoid content

The effect of ultrasound and different drying methods on the total carotenoid content (TCC) of purslane samples is given in Table 2. In this study, the TCC of control samples ranged from 37.70-67.93 mg/100 g and from 43.14-99.74 mg/100 g in the sonicated samples. The P4M5 samples showed higher total carotenoid content while the lowest was present in the P0M1 sample. The rise in carotenoid content may be due to breakdown of cell structure and inactivation of enzymes responsible for breakdown of carotenoids as a result of Sonochemical reaction caused by cavitation. Sonochemical reactions refer to chemical reactions induced by ultrasound waves, typically through the generation and collapse of cavitation bubbles, which create extreme temperatures and pressures. This process facilitates the formation of reactive species, such as hydroxyl radicals, that can modify the structure of phenolic compounds. The higher retention of total carotenoids in freeze drying may be due to the less degradation of these compounds. The ability to retain high levels of antioxidants and other bioactives makes freeze-dried products valuable for producing therapeutic formulations, such as herbal extracts or antioxidant-rich supplements. The results are supported by [36], who studied impact of different drying methods on acerola-ceriguela mixed pulp and reported higher retention of carotenoids in freeze dried acerola-ceriguela mixed pulp. Further, [9] showed that the pretreatments contributed to a shorter drying time by up to 40 % and a higher retention of carotenoid content.

### 3.6 Ascorbic acid content

The data for effect of ultrasound pretreatments and different dehydration methods on the ascorbic acid of purslane is shown in Table 2. The ascorbic acid (AA) of untreated dried samples was recorded between 302.36–398.60 mg/100 g while it ranged from 302.58-399.94 mg/100 g in the ultrasound pretreated samples. The highest ascorbic acid was present in the P4M5 sample. The lowest was recorded in P0M1. The elevated ascorbic acid content could be attributed to the sonication process in which little heat is provided and thus eliminates the dissolved oxygen through cavitation. Freeze-drying preserves ascorbic acid because the low processing temperature has little effect on the breakdown of water-soluble component. Also, it is conducted in a vacuum, significantly reducing exposure to oxygen. This minimizes oxidative reactions that can degrade sensitive bioactives like polyphenols and ascorbic acid. Nicolau-Lapena [28] reported an increase in the AA content when longer US treatment times were applied to plums at 30 kHz and 100 W. They increase of ascorbic acid was due to the elimination of entrapped oxygen due to cavitation, which is essential for AA degradation. Kittibunchakul *et al.*, [37] determined that convective hot-air drying showed lower retention of ascorbic acid compared with fruits dried using the freeze-drying process. Shofian *et al*., [38] reported no discernible (*p* > 0.05) difference in the ascorbic acid content of the fresh and freeze-dried fruits.

### 3.7 Ash and mineral content

The impact of ultrasound pre-treatments and different drying methods on the ash content of purslane samples was studied and the results are shown in Table 1. Different pretreatments and dehydration processes caused non-significant (*p* ≥ 0.05) changes in ash content in all the purslane samples. The ash content ranged from 18.09-18.84 % for untreated and for ultrasound pretreated samples, it ranged from 18.00-18.81 %. The high ash content of freeze-dried samples than other samples might be due to low moisture content. Kumar *et al*., [39] reported increase in drying temperature (40 − 70 °C) after pretreatment increased ash content compared to freeze-dried (control) sample.

The effect of ultrasound pre-treatment and different dehydration methods on the predominant minerals *viz*. potassium, magnesium and phosphorous of purslane samples was evaluated. The results are present in Table 3. It was observed that the potassium content of untreated dried purslane was between 487.12–491.20 mg/100 g and for ultrasound pretreated samples it ranged from 476.12-489.22 mg/100 g. The magnesium content of untreated dried samples was in range of 65.50–67.15 mg/100 g while it ranged from 57.15-67.15 mg/100 g in the samples which were pretreated with ultrasound following by drying. Similarly, the phosphorus of untreated dried purslane samples was in range of 41.2–43.44 mg/100 g while it ranged from 35.35-43.44 mg/100 g in the samples which were pretreated using ultrasound and then dried. The highest potassium, magnesium and phosphorus content (491.20, 67.15 and 43.44 mg /100 g) was found in P0M5 while the lowest mineral content was found in the P4M1 sample. The small variations in mineral content across different drying methods, suggest that temperatures had little or no effect on the mineral content of dried products. Because most minerals have limited volatility at high temperatures of up to 550–600 °C [40]. Bao *et al*., [41] reported that the collapse of cavitation bubbles generates an extremely high temperature and pressure environment, producing cracks on the surface of particles and expediting the mass transfer and diffusion of lixiviants, and thus, increasing the leaching efficiency of valuable metals. The findings are consistent with those of [42], who reported that drying amaranthus species and candied celeriac had no effect. The reduction in mineral content after ultrasound pretreatment could be due to leaching of minerals in US bath at the time of pretreatment. Because of US pretreatment, the cell structure is broken and leads to cavity formation. As a result, minerals are migrated through the cavity from the internal cell to the external zone.

**Table 3:** Effect of ultrasound (US) pretreatment and different methods on the Ash and Minerals of Purslane.

		P0	P1	P2	P3	P4
Ash (%)	M1	18.09 ± 0.31^aA^	18.06 ± 1.08^aA^	18.05 ± 1.30^aA^	18.03 ± 0.64^aA^	18.00 ± 1.06^aA^
	M2	18.31 ± 0.61^aA^	18.28 ± 1.57^aA^	18.26 ± 0.43^aA^	18.24 ± 0.09^aA^	18.21 ± 0.5^aA^
	M3	18.42 ± 0.33^aA^	18.40 ± 0.87^aA^	18.37 ± 0.47^aA^	18.34 ± 0.75^aA^	18.32 ± 1.14^aA^
	M4	18.5 ± 0.44^aA^	18.48 ± 1.18^aA^	18.46 ± 1.17^aA^	18.44 ± 0.42^aA^	18.40 ± 0.64^aA^
	M5	18.84 ± 1.29^aA^	18.81 ± 0.76^aA^	18.79 ± 0.90^aA^	18.76 ± 0.75^aA^	18.72 ± 0.83^aA^
	M6	18.72 ± 0.65^aA^	18.70 ± 1.22^aA^	18.68 ± 0.85^aA^	18.65 ± 0.26^aA^	18.62 ± 0.83^aA^
Mineral K(mg/100) Fw	M1	487.12 ± 1.03^dA^	485.14 ± 0.48^dB^	480.19 ± 0.75^dC^	478.2 ± 0.33^dD^	476.12 ± 0.85^cE^
	M2	487.93 ± 0.82^cdA^	485.94 ± 0.4^cdB^	480.95 ± 0.36^dC^	478.97 ± 0.53^dD^	476.89 ± 0.70^bcE^
	M3	489.5 ± 0.48^bcA^	487.52 ± 1.37^bcB^	482.56 ± 0.86^bcC^	480.58 ± 0.69^bcD^	478.49 ± 1.23^abE^
	M4	488.4 ± 0.46^cdA^	486.41 ± 0.85^cdB^	481.43 ± 0.99^cdC^	479.43 ± 1.04^cdD^	477.35 ± 0.08^bcE^
	M5	491.2 ± 0.79^aA^	489.22 ± 1.09^aB^	484.23 ± 0.48^aC^	482.24 ± 0.42^aD^	480.16 ± 1.25^aE^
	M6	490.18 ± 1.37^abA^	488.2 ± 0.72^abB^	483.21 ± 0.48^abC^	481.22 ± 0.93^abD^	479.14 ± 0.91^aE^
Mg(mg/100 g)Fw	M1	65.50 ± 0.98^aA^	64.12 ± 0.86^aA^	61.32 ± 1.03^bB^	59.65 ± 1.17^aB^	57.15 ± 0.48^aC^
	M2	65.94 ± 0.92^aA^	64.56 ± 0.91^aA^	62.06 ± 0.78^abB^	60.39 ± 0.97^aC^	57.85 ± 0.87^aD^
	M3	66.84 ± 1.14^aA^	65.46 ± 0.59^aA^	62.66 ± 0.82^abB^	60.99 ± 1.46^aB^	58.49 ± 1.46^aC^
	M4	66.14 ± 0.94^aA^	64.76 ± 1.46^aA^	61.96 ± 1.04^abB^	60.29 ± 0.31^aB^	57.79 ± 0.37^aC^
	M5	67.15 ± 0.84^aA^	65.77 ± 0.16^aA^	62.97 ± 0.78^aB^	61.3 ± 0.41^aC^	58.8 ± 1.26^aD^
	M6	67.00 ± 0.85^aA^	65.62 ± 0.64^aA^	62.81 ± 0.35^abB^	61.15 ± 1.03^aC^	58.65 ± 1.14^aD^
P (mg/100 g)	M1	41.12 ± 1.00^abA^	40.08 ± 0.52^bA^	39.12 ± 1.42^bAB^	37.56 ± 1.35^bB^	35.35 ± 1.12^bC^
	M2	41.86 ± 0.53^abA^	40.82 ± 1.05^abAB^	39.85 ± 0.81^abBC^	38.3 ± 1.07^abC^	36.09 ± 0.94^abD^
	M3	42.12 ± 0.59^abA^	41.08 ± 0.48^abAB^	40.12 ± 1.05^abB^	38.56 ± 0.65^abC^	36.35 ± 1.06^abD^
	M4	42.40 ± 0.85^abA^	41.36 ± 1.02^abAB^	40.4 ± 0.37^abB^	38.84 ± 0.09^abC^	36.63 ± 1.12^abD^
	M5	43.44 ± 0.31^aA^	42.4 ± 0.72^aAB^	41.44 ± 0.87^aB^	39.88 ± 0.74^aC^	37.67 ± 0.41^aD^
	M6	42.87 ± 0.3^abA^	41.83 ± 1.02^aAB^	40.87 ± 0.68^abB^	39.31 ± 0.6^aC^	37.1 ± 0.44^abD^

Values are expressed as Mean ± SD (n = 3), a-f within a column and A-F within a row, with different letters are significantly different. M1 (sun drying), M2 (solar tunnel drying), M3 (cabinet drying), M4 (vacuum drying), M5 (freeze drying) and M6 (microwave assisted drying). P0 (control), P1 (US treatment for 15 min), P2 (US treatment for 30 min), P3 (US treatment for 45 min), P4 (US treatment for 60 min).

### 3.8 Antioxidant activity

Table 4 shows the effect of ultrasound pre-treatments and different dehydration methods on the antioxidant activity of purslane. It was observed that the antioxidant activity of untreated dried purslane was less (62.21–87.15 %) as compared to treated samples (64.47–95.59 %). Hence our results showed that ultrasound pretreatment significantly (*p* ≤ 0.05) enhanced the DPPH free radical scavenging activity of the purslane samples. The highest DPPH percent inhibition was observed in the P4M5 while the lowest was found in P0M1 samples. The rise in antioxidant capability may be due to the rise in ascorbic acid and polyphenolic compounds that resulted from cavitation created during sonication which facilitates their extraction and enhanced availability of these components. Further, the sublimation process during freeze drying leaves a porous structure that retains the original morphology of the product. This can protect microstructures that house bioactive compounds, further enhancing their retention. Sun drying is most damaging drying method in comparison to other drying methods in terms of phenolic compounds and antioxidant activity. Kamiloglu *et al*., [43] reported that freeze-drying increased the total antioxidant capacity of dried fruits and vegetables, such as blueberry, raspberry, guabiju, red guava, muskmelon, watermelon, papaya and sweet potato because the low temperature in freeze drying prevents degradation of biologically active compounds. Similarly [44] found that sonication significantly improved antioxidant activity of apple juice. They reported that the percent inhibition of DPPH free radical increased from 39.71 up to 43.38 and 46.94 in all the samples sonicated for 30, 60 and 90 min, respectively, as compared to that (32.87) of control (non-sonicated) sample.

**Table 4:** Effect of ultrasound (US) pretreatment and different methods on the physicochemical, rehydration ratio, drying time of Purslane.

		P0	P1	P2	P3	P4
Antioxidant activity(% DPPH)	M1	62.12 ± 0.11^fE^	64.47 ± 0.06^fD^	65.17 ± 0.06^fC^	67.91 ± 0.08^fB^	68.7 ± 0.04^fA^
	M2	71.06 ± 0.05^eE^	73.62 ± 0.10^eD^	74.43 ± 0.08^eC^	79.11 ± 0.13^eB^	82.16 ± 0.07^dA^
	M3	79.11 ± 0.06^cE^	83.02 ± 0.09^cD^	84.6 ± 0.04^cC^	86.27 ± 0.01^cB^	89.96 ± 0.09^cA^
	M4	75.7 ± 0.15^dD^	76.93 ± 0.09^dC^	77.05 ± 0.09^dC^	79.39 ± 0.16^dB^	80.17 ± 0.09^eA^
	M5	87.15 ± 0.06^aE^	90.03 ± 0.04^aD^	92.91 ± 0.06^aC^	94.48 ± 0.07^aB^	95.59 ± 0.05^aA^
	M6	85.00 ± 0.11^bE^	88.38 ± 0.06^bD^	89.72 ± 0.1^bC^	90.01 ± 0.06^bB^	90.74 ± 0.1^bA^
L* value	M1	1.47 ± 0.10^fE^	2.31 ± 0.13^eD^	2.88 ± 0.07^eC^	5.67 ± 0.07^eB^	9.36 ± 0.04^eA^
	M2	1.83 ± 0.13^eE^	2.74 ± 0.11^dD^	3.85 ± 0.16^dC^	6.00 ± 0.13^dB^	9.69 ± 0.09^dA^
	M3	4.89 ± 0.08^cE^	5.23 ± 0.05^cD^	6.56 ± 0.10^cC^	16.47 ± 0.04^cB^	18.04 ± 0.05^cA^
	M4	2.07 ± 0.13^dD^	2.34 ± 0.04^eC^	2.10 ± 0.12^fD^	5.75 ± 0.07^eA^	3.22 ± 0.05^fB^
	M5	11.23 ± 0.02^aE^	11.81 ± 0.08^aD^	13.39 ± 0.09^aC^	16.65 ± 0.07^bB^	19.05 ± 0.06^aA^
	M6	5.89 ± 0.07^bE^	6.32 ± 0.10^bD^	7.80 ± 0.07^bC^	17.56 ± 0.11^aB^	18.50 ± 0.09^bA^
a* value	M1	−2.98 ± 0.10^bA^	−3.11 ± 0.04^cB^	−3.33 ± 0.12^bC^	−3.67 ± 0.07^bD^	−3.96 ± 0.15^bE^
	M2	−3.25 ± 0.08^cA^	−4.21 ± 0.07^dB^	−4.36 ± 0.05^cC^	−4.61 ± 0.06^cD^	−4.98 ± 0.03^cE^
	M3	−5.89 ± 0.11^dB^	6.61 ± 0.05^aA^	−6.81 ± 0.07^dC^	−6.89 ± 0.09d^CD^	−7.02 ± 0.07^dD^
	M4	0.49 ± 0.12^aE^	1.92 ± 0.08^bD^	3.36 ± 0.07^aC^	5.29 ± 0.13^aB^	8.51 ± 0.10^aA^
	M5	−8.69 ± 0.08^fA^	−10.45 ± 0.07^fB^	−10.62 ± 0.07^fC^	−10.88 ± 0.08^fD^	−11.01 ± 0.12^FdD^
	M6	−6.71 ± 0.06^eA^	−6.95 ± 0.07^eB^	−7.08 ± 0.08^eC^	−7.61 ± 0.07^eD^	−7.98 ± 0.02^eE^
b* value	M1	15.37 ± 0.55^cA^	14.53 ± 0.90^dAB^	14.17 ± 0.69^dB^	13.96 ± 0.86^dB^	12.03 ± 0.40^dC^
	M2	18.98 ± 0.07^bA^	17.55 ± 0.31^cB^	17.31 ± 1.14^cB^	17.10 ± 0.72^cB^	16.99 ± 0.50^cB^
	M3	19.41 ± 0.59^bA^	18.32 ± 0.23^bcB^	18.08 ± 0.87^bcB^	18.01 ± 0.80^bcB^	17.58 ± 1.03^bcB^
	M4	19.42 ± 0.63^bA^	19.33 ± 0.69^bA^	18.15 ± 0.86^bcB^	18.05 ± 0.74^bcB^	17.98 ± 1.02^bcB^
	M5	22.25 ± 0.92^aA^	21.35 ± 0.37^aAB^	21.12 ± 1.12^aB^	21.02 ± 0.44^aB^	20.97 ± 0.25^aB^
	M6	20.01 ± 0.308^bA^	19.25 ± 0.75^bAB^	19.06 ± 0.81^bAB^	18.44 ± 0.91^bB^	18.34 ± 0.86^bB^

Values are expressed as Mean ± SD (n = 3), a-f within a column and A-F within a row, with different letters are significantly different. M1 (sun drying), M2 (solar tunnel drying), M3 (cabinet drying), M4 (vacuum drying), M5 (freeze drying) and M6 (microwave assisted drying). P0 (control), P1 (US treatment for 15 min), P2 (US treatment for 30 min), P3 (US treatment for 45 min), P4 (US treatment for 60 min).

### 3.9 Color

Color is one of the most crucial factors in determining the quality of both processed and raw materials. This influences how well a product is received by customers [45]. The color (*L*, a* and b*) values of samples pretreated with ultrasound and dried by using different drying methods are illustrated in Table 4. The maximum *L* value (19.05) was observed in the P4M5 samples, while the minimum *L* value (1.47) was observed in P0M1. The maximum value of a* (−11.01) was observed in the P4M5 while the minimum value of a* (−2.98) was observed in P0M1. The maximum value of b* (22.25) was observed P0M5 while the minimum value of b* (12.03) was observed in P4M1 sample. Ultrasound pretreatment can inhibit enzymes such as polyphenol oxidase, responsible for browning, by disrupting cell membranes and inactivating these enzymes. This reduces enzymatic browning, which helps preserve the lightness (*L*) and the a* and b* values of the leaves. Our observations are well supported by [46], where they reported that freeze drying increased or maintained color characteristics in comparison with other drying treatments.

### 3.10 Total phenolic content

**Fig. 1:**
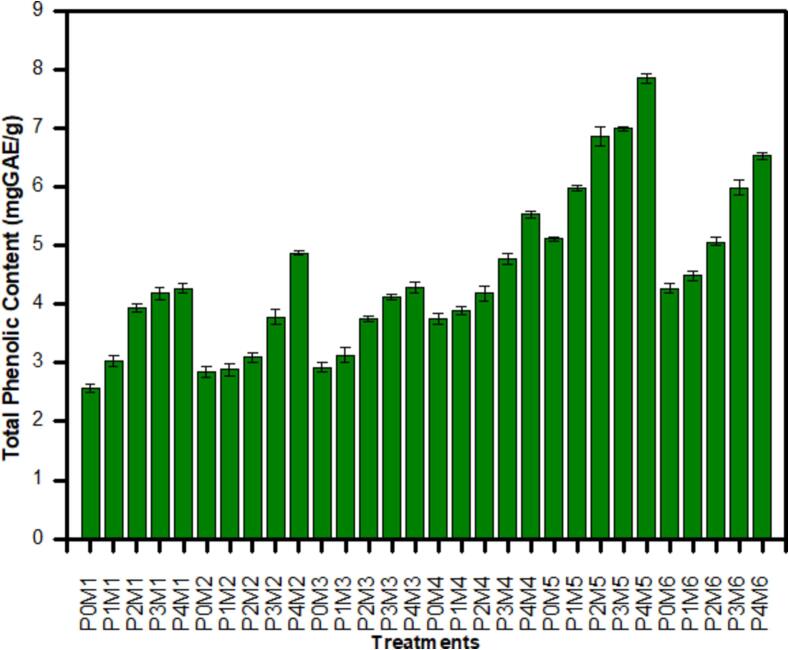
Effect of ultrasound pretreatment and different drying methods on TPC (mgGAE/g) of purslane.

The effect of ultrasound pre-treatments and different drying methods on the total phenolic content (TPC) of purslane is illustrated in Fig. 1. Different pretreatments and dehydration processes caused significant (*p* ≤ 0.05) changes in total phenolic content in all samples. It is evident from the Fig. 1 that TPC increased with the rise in ultrasound time. It was observed that the TPC of untreated dried purslane samples were in range of 2.57–5.11 mgGAE/g while it ranged from 2.73 to 7.85 mgGAE/g in the ultrasound pretreated samples. The highest TPC was recorded in the sample with P4M5 while the lowest was found in the P0M1 samples. The reason for decrease in phenolic content in non-sonicated sample may be due to their strong activity, get easily oxidized and degraded during drying. The highest content of TPC in freeze dried samples may be due to less damage to final products because of limited thermal and chemical degradation. Furthermore, generation of hydroxyl radicals through bubble collapse during ultrasound to the aromatic ring of phenolic compounds also causes their improvement in the purslane sample. The rapid freezing phase inhibits enzymatic reactions that might otherwise degrade bioactive compounds during slower drying methods. Our results are well supported by [37] and [44], who reported significant increase (P < 0.05) in total phenolics in juice samples sonicated for 30, 60 and 90 min as compared to non-sonicated juice sample. It ranged from 768 to 815 and 829 µg GAE/g respectively as compared to control (757 µg GAE/g).

### 3.11 Total flavanoid content

**Fig. 2:**
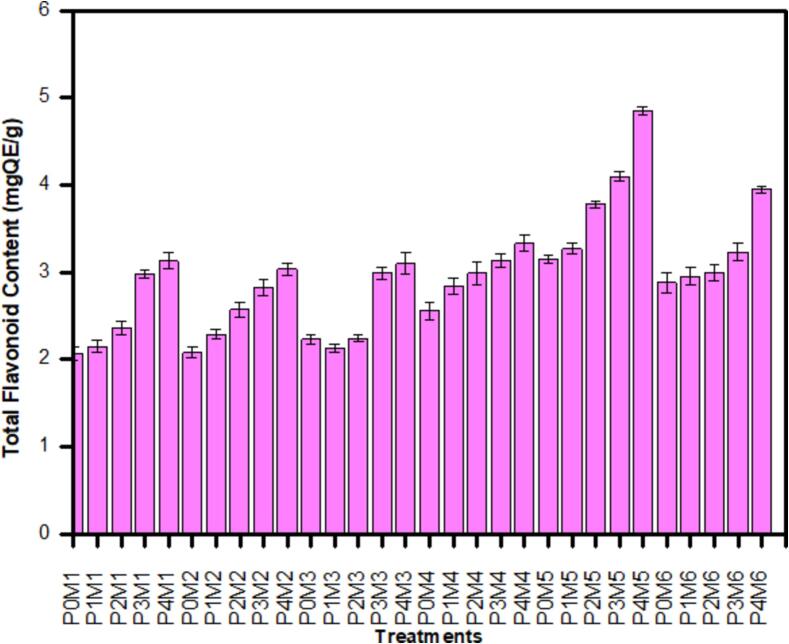
Effect of ultrasound pretreatment and different drying methods TFC (mgQE/g) of purslane.

The total flavanoid content (TFC) was calculated as quercetin equivalents (mgQE/g). The impact of ultrasound pre-treatments and different dehydration methods on the total flavanoid content (TFC) of purslane samples was studied and the results are shown in Fig. 2. The TFC was found higher 2.13–––4.85 mgQE/g in ultrasound treated purslane samples and lower 2.07–3.15 mgQE/g in case of non-sonicated samples. The highest TFC was present in the sample P4M5 samples while the lowest was recorded in the P0M1 samples. The highest content of TFC in freeze dried samples is because flavanoids are more vulnerable to high drying temperatures compared non-flavonoid phenolics, hence are protected at lower temperatures. While rise owing to ultrasonic pretreatment can be dedicated to giveout the bound form of the phenolic content due to rupture of the cell wall by the cavitation pressure. Moreover, the phenolic compounds' attachment to the aromatic ring may be the cause of the hydroxyl radicals (OH^−^) generated by sonication [47]. According to [48], ultrasonic treatment of kasturi lime and chokanan mango juice resulted in rise in the overall flavonoid content repectively. Our results are in accordance with the findings of [49], who studied the impact of freeze-drying and oven-drying methods on flavonoids content in two romanian grape varieties and observed that freeze dried grape skin retained more flavonoids content than hot-air dried. Abid *et al*., [44] also found significant increase (P < 0.05) in total flavonoids in sonicated apple juice when compared with non-sonicated juice. The increase in total flavonoids ranged from 486 to 600 and 607 µg/g of catechin equivalent compared to control which was 466 µg/g of catechin equivalent.

## 4 Conclusion

The findings of our study revealed that ultrasound pretreatment (60 min) followed by freeze drying is recommended for preserving purslane quality. The ultrasound pretreatment caused reduction in drying time, moisture content and water activity in each drying process. However, moisture content and water activity were lowest in the P4M5 samples while drying time was lowest in the P4M6. Total carotenoid content, ascorbic acid, chlorophyll content, total phenolic content, total flavanoid content, antioxidant activity, crude fat and rehydration ratio increased in the samples which were given ultrasound pretreatment and the highest was found in the P4M5 samples. Ultrasound pretreatment at 60 min, exhibited a significant influence on color attributes (L, a*, and b*) across various drying methods, with freeze drying resulting in the highest *L* and a* values. The mineral and ash content of purslane showed minimal variation across different drying methods and pretreatment processes, indicating stability under these conditions. Hence, this study provides remarkable insights into the synergistic effects of US-assisted pretreatment and drying methods. The integration of ultrasound with freeze-drying holds significant potential for industrial scalability, cost reduction, and environmental benefits. Its adoption in high-value industries, coupled with the use of renewable energy sources, can make it a sustainable and economically viable technology.

## CRediT authorship contribution statement

**Tajali Assad:** Writing – review & editing, Writing – original draft, Software, Formal analysis, Data curation, Conceptualization. **Zahida Naseem:** Writing – review & editing, Writing – original draft, Validation, Methodology, Formal analysis, Data curation, Conceptualization. **Sajad Mohd Wani:** Writing – review & editing, Writing – original draft, Software, Methodology, Investigation, Data curation, Conceptualization. **Aisha Sultana:** Investigation, Methodology, Project administration, Writing – review & editing. **Iqra Bashir:** Writing – review & editing, Resources, Project administration, Methodology, Data curation, Conceptualization. **Tawheed Amin:** Writing – review & editing, Software, Methodology, Investigation, Formal analysis. **Fauzia Shafi:** Writing – review & editing, Writing – original draft, Validation, Resources, Methodology, Investigation, Data curation. **B.S. Dhekale:** Writing – original draft, Supervision, Investigation, Formal analysis, Data curation, Conceptualization. **Imtiyaz Tahir Nazki:** Writing – review & editing, Writing – original draft, Supervision, Methodology, Formal analysis, Data curation. **Imtiyaz Zargar:** Writing – original draft, Software, Resources, Methodology, Investigation, Formal analysis. **A Raouf Malik:** Writing – review & editing, Visualization, Software, Resources, Investigation, Formal analysis, Data curation, Conceptualization. **Tawfiq Alsulami:** Writing – review & editing, Supervision, Methodology, Funding acquisition, Data curation, Conceptualization. **Robert Mugabi:** Writing – original draft, Supervision, Software, Methodology, Investigation, Funding acquisition, Formal analysis. **Gulzar Ahmad Nayik:** Writing – original draft, Validation, Supervision, Resources, Methodology, Investigation, Data curation, Conceptualization.

## Declaration of competing interest

The authors declare that they have no known competing financial interests or personal relationships that could have appeared to influence the work reported in this paper.
